# Adaptation and validation of an artificial intelligence based digital radiogrammetry tool for assessing bone health of indian children and youth with type-1 diabetes

**DOI:** 10.1007/s12020-023-03630-1

**Published:** 2023-12-21

**Authors:** Chirantap Oza, Misha Antani, Shruti Mondkar, Shital Bhor, Neha Kajale, Shilpa Kajale, Pranay Goel, Vaman Khadilkar, Anuradha Khadilkar

**Affiliations:** 1 Department of Paediatric growth and Endocrinology, Hirabai Cowasji Jehangir Medical Research Institute, Jehangir Hospital, Pune, India; 2 Consultant Paediatric Endocrinologist, Endogrow pediatric and adolescent endocrine centre, Ahmedabad, India; 3 Visiting consultant pediatric endocrinologist, Department of pediatrics, Narendra Modi Medical college, Ahmedabad, India; 4 Department of pathology, B.J. Medical college, Ahmedabad, India; 5 Interdisciplinary School of Health Sciences, Savitribai Phule University, Pune, India; 6 Consultant Radiologist, Department of radiology, Jehangir Hospital, Pune, India; 7 Department of Biology, Indian Institute of Science Education and Research Pune, Pune, India; 8 Senior Consultant, Jehangir Hospital, Pune, India

## Abstract

**Background and objectives:**

BoneXpert (BX) is an artificial intelligence software used primarily for bone age assessment. Besides, it can also be used to screen for bone health using the digital radiogrammetry tool called bone health index (BHI) for which normative reference values available are calculated from healthy European children. Due to ethnic difference in bone geometry, in a previous study, we generated reference curves based on healthy Indian children. The objectives of this study were: 1) To assess and compare bone health of Indian children with Type 1 diabetes (T1D) using both European and Indian BHI SDS reference data and 2) To identify determinants of poor bone health in Indian children and youth with T1D by using BHI tool (based on BHI-SDS Indian reference data) of BX.

**Method:**

The BHI was assessed retrospectively in 1159 subjects with T1D using digitalised left-hand x-rays and SDS were computed using European and Indian data. The demographic, anthropometric, clinical, biochemistry, dual x-ray absorptiometry (DXA) data and peripheral quantitative computed tomography (pQCT) data collection were performed using standard protocols and were extracted from hospital records.

**Results:**

The BHI correlated well with DXA and pQCT parameters in subjects with T1D. BHI-SDS calculated using Indian reference data had better correlation with height and DXA parameters. 8.6% study participants had low (less than −2) BHI-SDS (Indian), with height SDS having significant effect. Subjects with low BHI-SDS were older, shorter and had higher duration of diabetes. They also had lower IGF1 and vitamin D concentrations, bone mineral density, and trabecular density. Female gender, increased duration of illness, poor glycaemic control, and vitamin D deficiency/insufficiency were significant predictors of poor BHI-SDS.

**Conclusion:**

Our study highlights the utility of digital radiogrammetry AI tool to screen for bone health of children with T1D and demonstrates and highlights the necessity of interpretation using ethnicity specific normative data.

## Introduction

The International Society for Paediatric and Adolescent Diabetes (ISPAD) clinical practice consensus guidelines 2022 enlists poor bone health in ‘other complications’ and ‘associated conditions’ in children and adolescents with type 1 diabetes (T1D). It is suggested that bone mineral density (BMD), bone structure and fracture risk are altered in T1D [1]. Despite the higher risk of fractures, abnormal bone density as assessed by dual X-ray absorptiometry (DXA) is not always low in youth with T1D, however, decreased trabecular BMD has been demonstrated by peripheral Quantitative CT (pQCT) measurements [2, 3]. Although DXA is the recognised gold standard for assessing BMD in children by the International Society for Clinical Densitometry, in low and middle income countries like India, its availability in routine practice is limited [4]. Moreover, only relatively few pQCT scanners are available in hospitals and the lack of standardised paediatric references data for the micro-architectural and volumetric BMD parameters restrict its widespread use [5].

An older method of assessing bone health termed radiogrammetry was popular in 1960s wherein cortical thickness of the phalanges was measured manually in relation to their lengths, thereby producing an index of bone strength. BoneXpert (Visiana, Hørsholm, Denmark), an artificial intelligence (AI) tool primarily designed to assess bone age also performs radiogrammetry on digital X-rays, to provide the bone health index (BHI) [6]. BoneXpert (BX) uses the average values for cortical thickness (T), bone width (W) and bone length (L) of middle three metacarpals and then calculates BHI as πT(1 – T/W)/(LW)^0.33^. Normative BHI reference values calculated from a group of healthy European children stratified by gender and bone age are used to compute the standard deviation score (BHI-SDS) [7]. BHI has been reported to have strong correlation with DXA thereby making it a potential tool to assess bone health in children particularly with chronic illnesses like T1D [5].

It is well-known that for the correct interpretation of any anthropometric or bone health parameter in children, the use of age, gender, and ethnicity specific normative reference dataset is important. The average height and bone size of Southeast Asians including Indians is smaller than their western counterparts [8]. Thus, using European reference data for computing BHI-SDS in Indian children may be inappropriate. In a previous study, we produced BX derived BHI reference curves for 2–17-year-old Indian children using data on healthy subjects for assessment of bone health [manuscript accepted and in publication 10.1007/s00247-023-05824-1]. Thus, we conducted this study with the following objectives: 1) To assess and compare bone health of Indian children with T1D using both European and Indian BHI SDS reference data and 2) To identify determinants of poor bone health in Indian children and youth with T1D by using BHI tool (using BHI SDS Indian reference data) of BX.

## Methods

### Subjects and study design

We retrospectively reviewed medical records of children who had been seen at a tertiary care paediatric endocrine outpatient clinic between October 2015 and February 2022 and had at the same time point undergone anthropometric assessment, pubertal staging, a left-hand bone age X ray examination, biochemical testing and a DXA and pQCT scan. Records where patients had concurrent comorbidities like uncontrolled hypothyroidism, celiac disease, or disease duration less than 6 months (due to poor metabolic control in the period) were excluded. Thus, a total of 1159 radiographs (JPEG format) were analysed by BX to obtain BHI data. The BHI and BHI-SDS results were compared with the results of bone density and bone geometry assessments by DXA and pQCT. Biochemical assessments of bone metabolism parameters performed at the same time were also analysed.

Since deidentified data were used for this study, the ethics committee granted a waiver (dated 20^th^ July, 2022). An ethics approval was granted for earlier studies from which data have been extracted (Approvals dated- April 2016, Aug 2019, Sept 2020, Feb 2021). Parents of participants of earlier studies had given informed consent and children assent before any study procedures were performed.

Methodology used for collecting data is described in brief below.

### Clinical history and examination

Validated questionnaires had been used to obtain data on the age of the subjects, age at onset of diabetes, duration of diabetes, type of insulin regimen and total dose of insulin per day. Physical activity data were recorded using validated activity questionnaires adapted for Indian children [9].

### Anthropometry and pubertal assessment

Standing height was measured using a portable stadiometer (Leicester Height Metre; Child Growth Foundation, London, UK) to the nearest millimetre. Weight was measured to the nearest 100 g using an electronic scale. Body mass index (BMI) was computed by dividing weight in kilograms by height in metres squared. These anthropometric parameters were converted to Z-scores using Indian reference data [10]. Waist circumference (WC) was measured using World Health Organisation (WHO) guide to physical measurements and was converted to Z-scores using Indian reference data [11]. Pubertal assessment was performed by a trained paediatric endocrinologist using the rating scales of Tanner and Marshall [12].

### Blood pressure (BP)

BP was measured on the right arm with the child lying down quietly. The cuff was leak tested prior to commencement of the study. All air was removed from the cuff, the cuff was wrapped snuggly and neatly around the limb to allow one finger under the cuff. The cuff was placed 2–5 cm above the elbow crease. All the measurements were performed manually with the same oscillometric non-invasive BP device (Goldway™ Multipara Monitor—Model Number GS20).

### Automated assessment

The BHI obtained using BX version 3.2.2 was termed ‘raw BHI’. Corrected BHI was calculated using the formula BHI=raw BHI*(stature/(avL*50))^0.33333 where stature is height of subject and avL is average length of metacarpal bones. The BHI SDS was calculated using European reference data [13] and Indian reference data [manuscript in submission].

### Biochemical evaluation

A fasting blood sample (5 ml) was drawn between 7 and 9 am by a paediatric phlebotomist. Glycosylated haemoglobin (HbA1c) was measured by High-performance liquid chromatography (HPLC, BIO-RAD, Germany). Good glycaemic control was defined as HbA1c below 7% as per ISPAD guidelines [14]. The lipid profile (total cholesterol, triglycerides and HDL-C) was assessed using enzymatic method and low-density lipoprotein cholesterol (LDL-C) concentrations were calculated by the Friedewald formula [15]. Serum concentrations of total calcium were measured using a Calorimetric assay (ISA; AVL List GmbH, Graz, Austria). Creatinine was measured by the enzymatic method and phosphorous by the ultraviolet (UV) method. Intact serum PTH concentrations were measured by chemiluminescent immunoassay (Siemens, India; coefficient of variance = 3%). Alkaline phosphate (ALP) concentrations were measured using Star 21 semi-automatic biochemistry analyser by PNPP/AMP kinetic method. Serum IGF-1 concentrations were analysed by a solid phase enzyme-linked immunosorbent assay with an intra-assay coefficient of variation (CV) of 4.7% and inter-assay CV of 7.2%. Haemoglobin was estimated by spectrophotometry at a wavelength of 555 nm using a Horiba Yumizen H500 haematology analyser. The serum concentration of 25-hydroxy-vitamin-D3 (25OHD) were measured using radioimmunoassay (DiaSorin, Stillwater, MN, USA). The Indian Academy of Paediatrics Guidelines recommend that 25OHD concentrations of over 20 ng/ml are sufficient, between 12–20 ng/ml are insufficient and below 12 ng/ml are in the deficiency range in children and adolescents; we used these cut-offs to classify subjects as being sufficiency, insufficient or deficient [16].

### Dual X-ray absorptiometry

Areal BMD (aBMD) was measured using the Lunar iDXA (GE Healthcare, WI) fan-beam scanner (encore software – version 16). Sites measured were total body (TB) and lumbar spine [BMD [aBMD (g/cm^3^)]. The machine was calibrated daily, and service engineers regularly reviewed the calibrations. All scans and analyses were performed by the same operator. The CV for L1-L4 aBMD was 1%. Total body less head (TBLH) aBMD had a CV of 0.7%. Coefficients of variations for the iDXA parameters were computed from duplicate scans in children (*n* = 10) on the scanner where the study has been carried out. Z-scores for lumbar spine BMD (L1-L4 BMD) and TBLH aBMD, for age were computed using reference data for South Asian children in the UK. Height adjusted BMC was also used in analysis as aBMD is size dependent and Z-scores were not available under 4 years of age [17].

### Peripheral quantitative computed tomography

pQCT measurements were performed at radius using the Stratec XCT 2000 equipment (Stratec Inc., Pforzheim, Germany). All measurements were analysed by an integrated software for Stratec 2000, version 6.2. Images at 4% (proximal) and 66% (distal) length of radius were obtained during the scan. At 4% site of the radius using 0.59 mm voxel size, slice thickness –2.5 mm, contour mode 2, peel mode 2, trabecular volumetric BMD (vBMD) and bone mass were measured at a threshold of 180 mg/cm^3^. Cortical vBMD, cortical thickness, periosteal circumference, endosteal circumference, and stress strain index (SSI), were measured at 66% of the radius using a threshold of 711 mg/cm^3^, contour mode 3. The coefficient of variation for the pQCT bone parameters from duplicate scans (*n* = 10) ranged from 0.65 to 3%. The CV for the total, trabecular, and cortical density was 0.7, 3.0, and 0.8% respectively. The cortical thickness measurements had a CV of 2.6%.

### Statistical analysis

All statistical analyses were carried out using the SPSS for Windows software program, version 26 (SPSS, Chicago, IL, USA). All variables were tested for normality before performing statistical analyses. We assessed the correlation between the BHI-SDS (Indian and European reference data) with anthropometric, DXA and pQCT derived parameters using the Spearman correlation coefficient. Differences in means were tested using Student’s t test for parametric data and Mann Whitney U test for non-parametric data. The influence of height SDS, weight SDS, BMI SDS and puberty on BHI SDS was assessed using a stepwise regression analysis. A binary logistic regression was performed to test the relationships between dichotomous-dependent variables and continuous predictors. The dependent variable in the model was low BHI-SDS (less than −2) while the independent variables were gender, duration of diabetes, insulin requirement, glycaemic control and vitamin D levels. Statistical significance was set at *p* < 0.05.

## Results

**Fig. 1:**
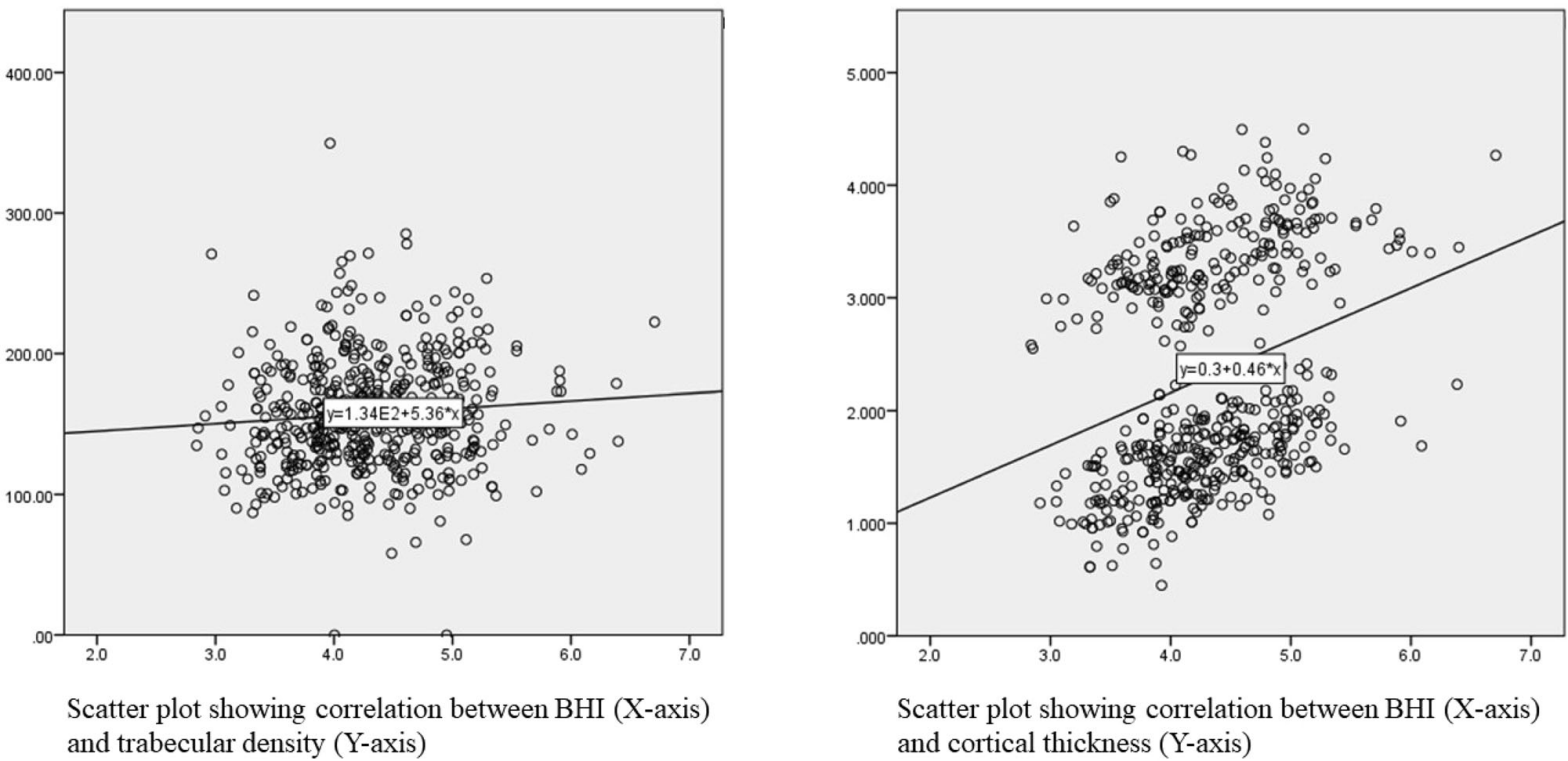
Scatter plot showing correlation between BHI and pQCT parameters

Of the total 1159 records analysed, 550 (47.5%) were male and 609 (52.5%) were females. The mean age of participants whose data were included in the study was 11.5 ± 3.9 years (range 1–25 years) with a mean duration of diabetes of 4.4 ± 3.4 years. The mean HbA1c was 10.0 ± 2.1% and mean insulin requirement was 1.0 ± 0.3 IU/kg/ day. All the subjects were on basal-bolus regimen. Only forty-eight (4.2%) subjects met the glycaemic control target. 438 (37.8%) subjects were prepubertal while of the rest, 446 (38.5%) were pubertal and 275 (23.7%) were post-pubertal. The mean BHI for the cohort was 4.0 ± 0.6 (1.6–6.7). The BHI had significant positive correlation with lumbar spine BMD (0.69). As shown in Fig. 1, the BHI also had significant positive correlation with pQCT parameters like trabecular density (0.38) and cortical thickness (0.36).

We report a correlation of 0.8 (*p* < 0.05) between BHI-SDS computed using European (BHI-SDS-E) and Indian (BHI-SDS-I) reference data. The descriptive data on BHI-SDS computed on the 1159 X rays using European and Indian normative data is illustrated in Table 1A. The paired sample t-test showed that mean BHI-SDS-I was significantly higher than BHI-SDS-E. The correlation of BHI-SDS (European and Indian) with height and DXA parameters is illustrated in Table 1B. As seen in Table 1B, only the BHI-SDS-I had significant positive correlation with height and bone mineral content. Moreover BHI-SDS-I had stronger significant positive correlation with BMD as compared to BHI-SDS-E. Thus, BHI-SDS-I may be more suitable for use in Indian children and henceforth, we have used Indian reference data for computation of BHI-SDS.

**Table 1:** A: Descriptive data of BHI SDS using Caucasian and Indian normative data. B: Correlation of BHI SDS with height and DXA parameters

Parameter	BHI SDS Caucasian	BHI SDS Indian
Mean^a^	−2.2	−0.2
Standard Deviation	1.3	1.3
Maximum	3.3	4.9
Minimum	−8.4	−5.8
Proportion with BHI SDS < −2	57.9%	8.6%

Parameter	BHI SDS European	BHI SDS Indian
Height	−0.047	0.060^b^
L1-L4 BMD	0.122^b^	0.135^b^
L1-L4 BMC	0.046	0.098^b^
L1-L4 Area	−0.0180	0.062
TBLH BMD	0.144^b^	0.190^b^
TBLH BMC	0.037	0.117^b^
TBLH Area	−0.058	0.051

*BMD* bone mineral density, *BMC* bone mineral content, *TBLH* total body less head

^a^Statistically significant difference *p* < 0.05 on performing paired t-test

^b^Statistically significant at *p* < 0.05

**Fig. 2:**
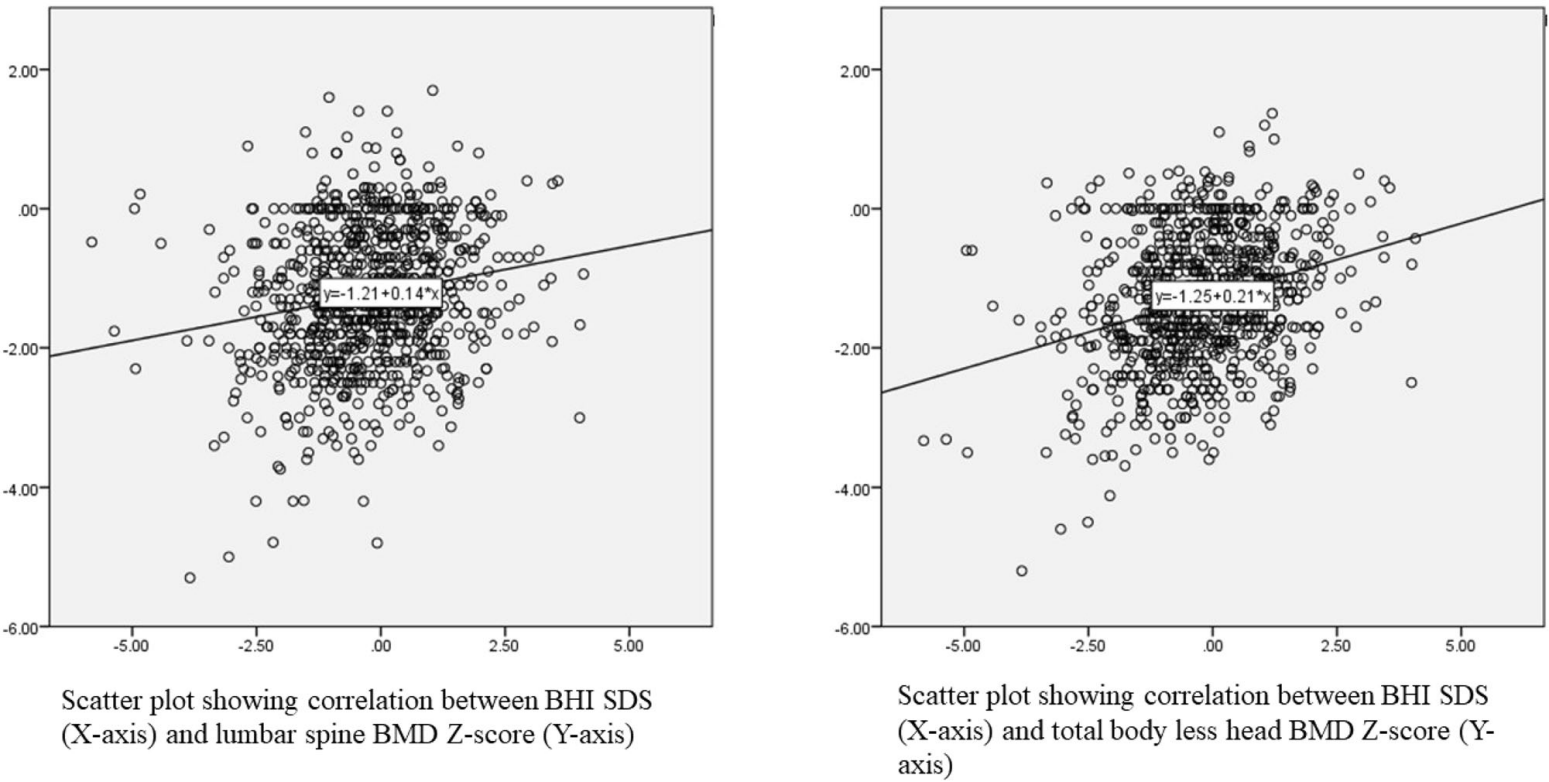
Scatter plot showing correlation between BHI-SDS and DXA parameters

The correlation of BHI-SDS-I with L1-L4 BMD Z-score and TBLH BMD Z-score was also significant as shown in Fig. 2. The BHI-SDS-I was independent of age (*r* = −0.03), weight (*r* = 0.01), BMI (*r* = −0.02) and pubertal stage (*r* = 0.03). In a stepwise regression analysis, using two models containing either height SDS, weight SDS and pubertal status or BMI SDS and pubertal status, only height SDS was shown to have a significant but small effect on BHI-SDS-I (*r*^2^ = 0.034).

We report that 57.3% (*n* = 664) had BHI-SDS-I values below the mean for age- and sex-matched healthy children. In 8.6% (*n* = 100) subjects, we found significantly decreased BHI-SDS-I values (less than 2 SD). The comparison of clinical, biochemistry, DXA and pQCT parameters of T1D subjects with poor BHI-SDS-I as compared to normal BHI-SDS-I is shown in Table 2. Subjects with low BHI-SDS-I were older, shorter and had higher duration of illness. They also had lower IGF1 values, vitamin D levels, BMD, and trabecular density. As shown in Table 3, on performing the binary logistic regression, we report female gender, higher duration of illness, poor glycaemic control, and vitamin D deficiency/insufficiency as significant predictors of poor BHI-SDS-I.

**Table 2:** Comparison of clinical, biochemistry, DXA and pQCT parameters of T1D subjects with low BHI-SDS-I (<−2) as compared to BHI-SDS-I within reference range (>−2)

Parameter	Normal BHI-SDS-I	Low BHI-SDS-I
	Mean ± Std. Deviation	Mean ± Std. Deviation
Clinical		
Chronological age in years^a^	11.5 ± 4	12.8 ± 4.2
Duration of illness in years^a^	4.4 ± 3.3	6.2 ± 4.3
Height Z-score^a^	−0.8 ± 1.1	−1.5 ± 1.5
Weight Z-score	−0.7 ± 1.1	−0.9 ± 1.4
Body mass index Z-score	−0.4 ± 1	−0.2 ± 1.2
Waist Z-score	−1.3 ± 1.1	−1.3 ± 1.1
Waist hip ratio	0.9 ± 0.1	0.9 ± 0.1
Total moderate to vigorous activity daily in minutes	62.8 ± 39.7	56.1 ± 33.2
Systolic blood pressure in mmHg	105.1 ± 11.2	106.5 ± 13.5
Diastolic blood pressure in mmHg	68.5 ± 9.5	70.2 ± 12
Insulin requirement in U/kg/day	1.1 ± 0.4	1.2 ± 0.4
Biochemistry		
Creatinine in mg/dL	0.8 ± 2.1	0.8 ± 0.3
HbA1c %	10.1 ± 2.1	10.3 ± 2.7
IGF1 in ng/ml^a^	166.4 ± 96.5	131 ± 72.4
PTH in pg/mL	53.8 ± 49.1	59.4 ± 55.4
Vitamin D in ng/mL^a^	19.5 ± 11.8	15.8 ± 8.2
Alkaline phosphatase in IU/L	248.7 ± 110.8	236.8 ± 101.6
Serum Calcium in mg/dl	9.5 ± 1.1	9.4 ± 0.6
Serum Phosphorus in mg/dl	4.1 ± 2.2	3.9 ± 0.8
Haemoglobin in g/dL	13.1 ± 1.7	12.9 ± 1.9
Cholesterol in mg/dl	145 ± 36.3	147.6 ± 38.5
HDL in mg/dl	48.8 ± 12.3	47.7 ± 12.6
LDL in mg/dl	79.1 ± 33.2	79.2 ± 30.7
Triglyceride in mg/dl	81.1 ± 68.4	99.7 ± 111
VLDL in mg/dl	16.4 ± 13.6	20 ± 22.2
DXA		
L1-L4 BMD in gm/cm^2^	0.8 ± 0.2	0.8 ± 0.3
L1-L4 BMC in gm	25.5 ± 12	26.5 ± 12.6
L1-L4 Z-score^a^	−1.3 ± 1	−1.8 ± 1.3
TBLH BMD in gm/cm^2^	0.7 ± 0.2	0.7 ± 0.2
TBLH BMC in gm	863.7 ± 390	893.7 ± 438.4
TBLH Z-score^a^	−1.3 ± 1	−1.9 ± 1.3
LSBMAD in gm/cm^3^	0.1 ± 0.1	0.1 ± 0.1
pQCT		
SSI in mm cube	158.6 ± 58.4	155.9 ± 62.8
Trabecular Area in mm square	103.4 ± 27.9	110.7 ± 29.6
Trabecular Density mg per cm cube^a^	159.3 ± 39.8	139 ± 37.1
Cortical Area in mm square	48.1 ± 12.9	45.6 ± 13.8
Cortical Density in mg per cm cube	1057.7 ± 63.9	1060.7 ± 60.6
Cortical thickness in mm	2.4 ± 1	2.3 ± 1

*HbA1c* glycosylated haemoglobin, *IGF1* insulin like growth factor-1, *PTH* parathyroid hormone level, *HDL* high density lipoprotein, *LDL* low density lipoprotein, *VLDL* very low density lipoprotein, *BMD* bone mineral density, *BMC* bone mineral content, *TBLH* total body less head, *LSBMAD* lumbar spine bone mineral apparent density, *SSI* stress strain index

^a^Statistically significant difference between two groups at *p* < 0.05

**Table 3:** Binary logistic regression for low BHI-SDS-I (<−2) in relation to various variables

Parameter	B	S.E.	Wald	df	Significance	Exp(B)
Gender*	0.53	0.23	4.95	1	0.02	1.7
Duration of illness*	0.13	0.02	20.6	1	<0.01	1.14
Insulin requirement	−0.11	0.33	0.11	1	0.73	0.89
Glycaemic control categories*	1.00	0.44	5.04	1	0.02	2.72
Vitamin D categories*	0.57	0.26	4.72	1	0.03	1.77
Constant	−3.76	0.46	66.45	1	0	0.02

* Statistically significant parameter at *p* < 0.05

## Discussion

We found that BHI correlated well with DXA and pQCT parameters in Indian subjects with T1D. We also report that BHI-SDS calculated using Indian reference data had better correlation with height and DXA parameters and thus may be more suitable to screen for bone health of Indian children and youth. 8.6% study participants had low BHI-SDS-I (less than −2), with height SDS having significant effect on BHI-SDS-I. Subjects with low BHI-SDS-I were older, shorter and had higher duration of diabetes. They also had lower IGF1 values, vitamin D levels, DXA calculated lumbar spine and total body less head BMD Z-scores, and pQCT assessed trabecular density. Female gender, increased duration of illness, poor glycaemic control, and vitamin D deficiency/insufficiency were significant predictors of poor BHI-SDS-I.

We have thus adapted the BHI tool of the BoneXpert for use in Indian children. An example of the application of use of BHI SDS for assessment of bone health in Indian children is illustrated in Appendix A.

The correlation of BHI with DXA and pQCT in children with T1D is comparable to that in healthy children [18]. A significant positive correlation of BHI with lumbar spine BMD and trabecular density at radius was reported in healthy children (*r* = 0.7 and 0.21 respectively) which is similar to our study (*r* = 0.69 and 0.11 respectively). As the BHI measures cortical thickness, the trabecular indices obtained from pQCT tend to correlate less closely with BHI-SDS than cortical density. Height is shown to have a small effect on BHI-SDS in both healthy children as well as subjects with T1D [18]. Our study demonstrates that BHI computed by BX largely correlates with the DXA and pQCT readings as in healthy children and thus, maybe a useful tool to screen bone health in children with T1D sparing risk of additional irradiation.

We report significantly lower BHI-SDS as per European reference data in comparison to Indian normative data in Indian children and youth with T1D. This may be attributed to ethnic differences. A study on differences in bone mineral in young Asian and Caucasian Americans showed that Asian and Caucasian cohorts differed in body size, diet, and physical activity. The differences in BMD between Asian and Caucasian subjects were largely attributable to differences in weight, pubertal stage, and weight-bearing activity. Authors concluded that observed differences in bone mineral between Asians and Caucasians may be attributed to the smaller bone size of Asians [19]. We thus suggest that using ethnicity specific (Indian) BHI reference data for assessment of bone health reduces the number of false positives and may help to reduce the burden of assessing poor bone health in children and youth with T1D in resource-limited settings.

To the best of our knowledge, only one study prior to ours has assessed BHI of subjects with T1D. They report 20.37% of 54 patients, with BHI-SDS values less than 2 SD. Similar to our study they also reported lower IGF-1 levels and higher duration of diabetes in subjects with low BHI SDS. However, unlike our study, they report significantly lower phosphorous levels in subjects with lower BHI SDS [20]. The difference in results may be attributed to larger sample size and use of ethnicity specific SDS scores in our study.

Various studies have described contrasting results regarding determinants of poor bone health in children with T1D. While some studies have shown no role of glycaemic control in BMD in subjects with T1D [2], some have suggested that poor glycaemic control was associated with low BMD and concluded that optimal glycaemic control may prevent low BMD and altered bone turnover in T1D and decrease fracture risk. High levels of advanced glycated end-products of collagen directly inhibit osteoblast function by changing the bone cell-matrix interactions while lower collagen strength may also result from glycosylation of collagen [21].

We also found that the trabecular, and not the cortical density was significantly lower in subjects with low BHI-SDS vs those with normal BHI-SDS. In an earlier study, we have reported that trabecular bone was more severely affected in patients with T1D and that it was negatively corelated with glycaemic control as judged by HbA1c [22].Further, studies in adults with type 2 diabetes also suggest a negative corelation of trabecular density with glycaemic control [23]. Leslie et al report that although subjects had higher overall bone density, the trabecular bone score was lower in subjects with diabetes [24]. Higher density and lower porosity of cortical bone versus larger remodelling area and more metabolically active tissue in the trabecular bone are suggested reasons for trabecular bone being more affected [25].

Further, conflicting data on bone mineral characteristics by gender for children with T1D are reported. A recent study similar to ours reports female gender to be linked with low BMC as androgen promotes periosteal bone formation, while oestrogen inhibits or has no effect on it. The same study also reports optimum serum IGF1 concentrations are necessary for normal bone growth and density in children with T1D [26]. Low concentrations of IGF-1 has a negative effect on bone and is associated with poor bone health in patients with T1D as it plays a critical role in osteoblast differentiation, collagen deposition, and bone mineralisation [27].

Associations between vitamin D and BMD have not been established thus far, although they are still being investigated. However, a review in children with vitamin D concentrations below 35 nmol/L reports an increase in total body BMC and lumbar spine BMD of ∼2% over a follow-up period of 1–2 years post supplementation [28]. A review on impact of T1D on bone health in children concluded that low bone turnover status in children with T1D can be explained by several mechanisms including high prevalence of vitamin D deficiency and suggested vitamin D supplementation as one of the standards of care [29].

To the best of our knowledge, ours is the first study to objectively report the role of an AI based tool such as BHI of BX in assessing bone health of children with T1D in comparison to standard methods like DXA and pQCT. Ours is also the first study to validate and demonstrate the use of ethnicity specific BHI-SDS to assess bone health of children using digital radiogrammetry. Cross-sectional design, lack of longitudinal follow-up, lack of data on fractures and data from single centre are the limitations of our study.

In conclusion, our study highlights the utility of digital radiogrammetry AI based tool to screen for bone health of children with T1D and demonstrates and highlights the necessity of interpretation using ethnicity specific normative data. Ethnicity-specific BHI-SDS has the potential to be a useful marker to assess bone health in children and adolescents with type 1 diabetes. However, there is a need for longitudinal studies to assess its correlation to fracture.

## Appendix A

Examples of calculations of Z scores

Case 1: A 15.7 years old girl with T1D had height 160 cm had raw BHI 4.7, lumbar spine BMD 1.1 g/cm^2^, BMC 51.3 g and BA 46.5 cm^2^. The lumbar spine BMD Z-score was −0.2. The corrected BHI using equation for JPEG images was 4.6. Using Indian reference data this girl was not short (Z-score= 0.3). Using percentile curves and LMS values represented by Indian data, the BHI SDS was −0.2 while BHI-SDS using European reference data was −1.9.

Case 2: 11.6-year-old boy with T1D had height 135.7 cm, raw BHI 3.5, lumbar spine and total body less head BMD were 0.1 g/cm^2^ and 0.5 g/cm^2^ respectively. The lumbar spine BMD Z-score and total body less head BMD Z-score were −1.8 and −2.4 respectively. The corrected BHI using equation for JPEG images was 3.3. Using Indian reference data this boy was short (Z score = −2.0). Using percentile curves and LMS values represented by Indian data, the BHI-SDS was −1.8 while BHI-SDS using European reference data was −3.5.
